# P21 Promoting antimicrobial stewardship and sustainability utilizing a ‘Go Green’ IV to oral switch (IVOST) policy and electronic prescribing prompts in antimicrobials with high oral bioavailability

**DOI:** 10.1093/jacamr/dlad066.025

**Published:** 2023-06-14

**Authors:** R Rodger, S Thompson, A Robertson, C Pender, G Ray, L Chisholm

**Affiliations:** Royal Alexandra Hospital, NHS Greater Glasgow & Clyde; Royal Alexandra Hospital, NHS Greater Glasgow & Clyde; Royal Alexandra Hospital, NHS Greater Glasgow & Clyde; Royal Alexandra Hospital, NHS Greater Glasgow & Clyde; Royal Alexandra Hospital, NHS Greater Glasgow & Clyde; Royal Alexandra Hospital, NHS Greater Glasgow & Clyde

## Abstract

**Background:**

Antimicrobial stewardship (AS) is crucial in reducing antimicrobial resistance (AMR) and the sustainability impact of inappropriate antimicrobial use.^1^ Promotion of the oral route and IV to oral switch (IVOST) are key AS initiatives providing benefits for patients, staff and the environment including reduced risk of line infections and AMR, reduced workload and costs, reduced plastic waste and improved sustainability.^2^ A recent quality improvement (QI) initiative to promote oral metronidazole (bioavailability > 90%) in eligible surgical patients at the Royal Alexandra Hospital (RAH) resulted in a change in prescribing behaviour and a 45% median reduction in IV metronidazole use.^3^

**Objectives:**

To scale and spread promotion of the oral route in antimicrobials with high oral bioavailability with the aim of reducing collective IV use by 20% by February 2023 at RAH.

**Methods:**

A QI approach was used to promote the oral route where appropriate in the following agents: metronidazole, levofloxacin, fluconazole, ciprofloxacin, co-trimoxazole, clindamycin, linezolid and rifampicin. Techniques included the following: ‘Go Green IVOST’ posters in key ward areas highlighting benefits of the oral route; targeted pharmacist and pharmacy technician prospective audit and feedback using the electronic prescribing system; presentations to ward and pharmacy teams; staff champions and electronic IVOST prompts displayed when prescribing and administering the targeted agents IV. To measure improvement, oral and IV usage data were measured at baseline and for 24 months post change. Data were displayed on run charts to determine if the QI target was achieved.

**Results:**

There was a 44% median reduction in IV defined daily doses (DDDs) in the targeted antimicrobials, surpassing the 20% target (Figure 1). There was a median 10% shift in the proportion of these agents given IV as a percentage of total (IV plus oral) use (Figure 2) The greatest reduction (60%) was in IV metronidazole where usage declined from a monthly baseline of 667 to 267 DDDs. This equates to a median reduction of 1200 IV metronidazole administrations and nearly 600 hours nursing time saved per month. IV fluconazole and IV levofloxacin usage declined from baseline by a median of 35% and 24%, respectively.

**Conclusions:**

A QI approach to promote appropriate use of the oral route in antimicrobials with high oral bioavailability resulted in a change in prescribing behaviour and a sustained reduction in IV prescribing of these agents. This is important in terms of improved AS, patient care and safety, healthcare cost and efficiency and environmental sustainability.

**Figure 1.**
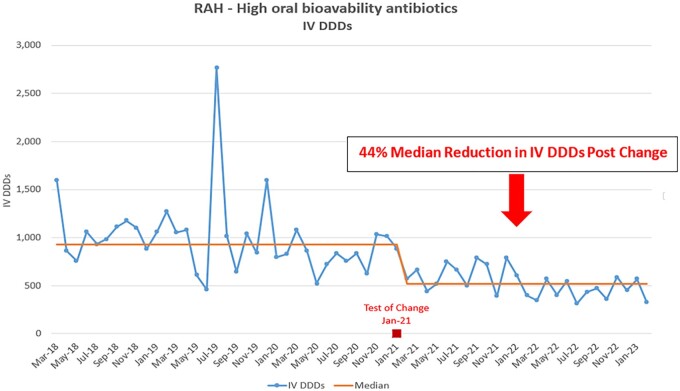
IV DDDs.

**Figure 2.**
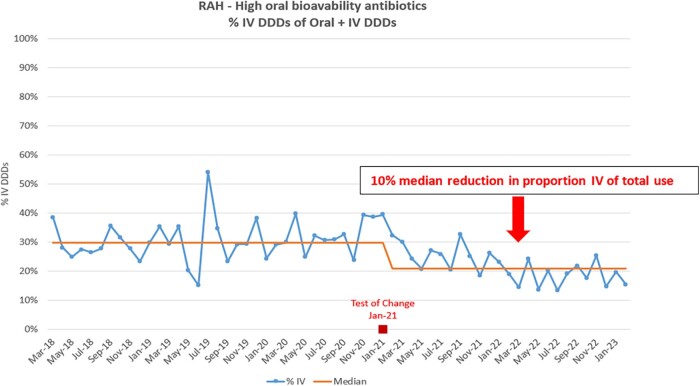
Percentage IV DDDs of oral + IV DDDs.

## References

[1] Antimicrobial Resistance Collaborators . Global burden of bacterial antimicrobial resistance in 2019: a systematic analysis. Lancet 2022; 399: 629–655. 10.1016/S0140-6736(21)02724-0 35065702 PMC8841637

[2] Scottish Government . Scottish Management of Antimicrobial resistance Action Plan 2014–18 (ScotMARAP 2). Scottish Government; 2014.

[3] Rodger R , Robertson A, McGee C et al P03 Improving antimicrobial stewardship and sustainability by promoting oral metronidazole in eligible surgical patients at The Royal Alexandra Hospital. JAC Antimicrob Resist 2022; 4(Suppl 2): dlac053.003.

